# Anteverted Concha: Case Series, Proposed Classification, Novel Management Technique, and Literature Review

**DOI:** 10.1055/s-0044-1801807

**Published:** 2025-02-24

**Authors:** Chandrashekhar Subhash Chalwade, Vidhi M. Mehta, Senthil Kumar R.M, Vinita Puri

**Affiliations:** 1 Department of Plastic Surgery, Seth G.S Medical College and K.E.M Hospital, Mumbai, Maharashtra, India

**Keywords:** ear auricle, congenital ear anomaly, anteverted concha, reverse concha, classification.

## Abstract

**Background** An anteverted concha is an uncommon ear anomaly that causes anterior ear deformity and falls under auricular deformation. Treatments vary from conservative to surgical. Limited global case reports may be due to under-recognition, prompting a proposed classification. Patients with an anteverted concha were evaluated in our department and a classification system was formulated.

**Materials and Methods** This study was conducted in a plastic surgery department at a tertiary care teaching hospital over a period of 2 years, from January 2021 to December 2022. This study included 22 ears of 16 patients. One patient underwent surgical correction, and two patients underwent molding therapy with excellent outcomes. In addition to reporting these patients' outcomes, we reviewed their cases to propose a classification system. Additionally, an in-depth review of the literature on the reported cases was performed.

**Results** Molding therapy during the early neonatal period and surgical correction later in life yielded excellent outcomes.

**Conclusion** An anteverted concha may be underdiagnosed. Correction is typically straightforward and may not necessitate surgery if it is diagnosed early. Surgical correction leads to excellent outcomes in symptomatic anteverted conchae in adulthood.

## Introduction

An anteverted concha, also known as inverted concha, is an uncommon anomaly characterized by anterior convexity of the concha, resulting in an aesthetically noticeable deformity of the external ear. 1 2 It has been previously reported as a convex 3 or reverse 4 conchal deformity.

When classifying external ear anomalies, they are traditionally categorized as either malformations or deformations. 5 Auricular malformations are abnormalities that arise from inherent developmental issues and typically occur between the fifth and ninth weeks of gestation. 6 These malformations can involve deficiencies or excesses in various components of the auricle. In contrast, auricular deformations occur when external forces disrupt the normal development of the ear structure and can occur at any point during or after gestation. 7

An anteverted concha falls into the category of auricular deformations and can manifest either unilaterally or bilaterally. 3 4 8 9 Many of the patients are asymptomatic except a few who experience functional issues. These include conductive hearing loss, cerumen collection and hearing aid or earphone retention issues. 8 The management options for this condition span from conservative measures, such as splinting or molding, 1 7 10 11 12 13 to various surgical interventions. 3 4 8 9 14

In this study, we present a small case series of anteverted conchae with management for those wishing to correct it. There have been very few reported cases of anteverted conchae globally, and to the best of our knowledge, there is currently no classification system for this anomaly in the English literature. Here, we share our experience in managing patients with anteverted conchae and propose a classification system for this particular type of ear anomaly. We believe that this deformity is likely to be underdiagnosed, and this study will help further its awareness, detection, and standardization of management strategies.

## Materials and Methods

This study was conducted in the plastic surgery department at a tertiary care teaching hospital over 2 years, from January 2021 to December 2022. The authors assert that all procedures contributing to this work complied with the ethical standards of the relevant national and institutional guidelines on human experimentation and the Helsinki Declaration of 1975, as revised in 2008. Approval was obtained from the institutional ethics committee.

This was a retrospective, qualitative case series of patients who presented with features of an anteverted concha. Patients who provided consent and had anteverted conchae were included in this study. Patients with posttraumatic or postinterventional ear deformities or those who were unwilling to participate in the study were excluded. A total of 22 ears from 16 patients were included in the study over a duration of 2 years (January 2021 to December 2022). The aim of this study was to analyze anteverted concha cases, prepare a possible classification and an algorithm for its management, and perform a review of the literature.

**Fig. 1:**
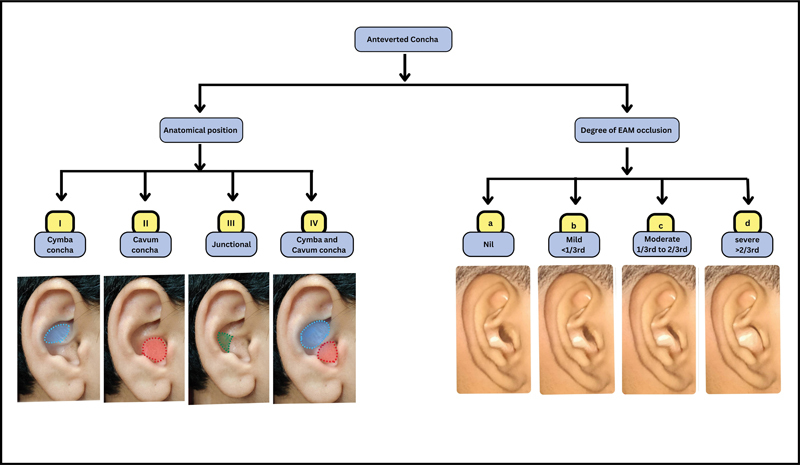
Classification of anteverted concha depending on anatomical involvement and degree of external auditory meatus (EAM) occlusion.

Demographic data, presenting symptoms, and clinical findings were recorded, analyzed, and tabulated ( Table 1 ). After analyzing the data, we formulated a classification system ( Fig. 1 )

**Table 1:** Clinical and demographic data of patients with AVC presented to our department

Sl. no.	Patient	Age	Gender	Side	Type	Clinical presentation	Intervention
1	1	19 y	Male	R	IIc	Moderate hearing loss, cerumen collection	Surgical excision with posterior mobilization
				L	IIc	Nil	No intervention
2	2	35 y	Male	R	IIa	Difficulty in wearing earphones	No intervention
				L	IIa	Difficulty in wearing earphones	No intervention
3	3	9 y	Male	R	IIb	Nil	No intervention
				L	IIb	Nil	No intervention
4	4	6 y	Male	R	Normal ear	Nil	No intervention
				L	IIIa	Nil	No intervention
5	5	29 y	Male	R	Normal ear	Nil	No intervention
				L	IIIa	Difficulty in wearing earphones	No intervention
6	6	4 y	Male	R	Ia	Nil	No intervention
				L	Normal	Nil	No intervention
7	7	8 y	Male	R	Normal	Nil	No intervention
				L	IIIa	Nil	No intervention
8	8	23 y	Female	R	Normal	Nil	No intervention
				L	IIb	Aesthetic	No intervention
9	9	2 d	Male	R	IIb	Aesthetic	Splinting and molding
				L	IIb	Aesthetic	Splinting and molding
10	10	27 y	Female	R	IIa	Nil	No intervention
				L	Normal	Nil	No intervention
11	11	2 d	Female	R	IIb	Aesthetic	Splinting and molding
				L	IIc	Aesthetic	Splinting and molding
12	12	23 y	Female	R	IIa	Nil	No intervention
				L	IIb	Aesthetic	No intervention
13	13	32 y	Male	R	Normal	Nil	No intervention
				L	IIc	Nil	No intervention
14	14	17 y	Male	R	Normal	Nil	No intervention
				L	IIb	Aesthetic	No intervention
15	15	19 y	Male	R	IIc	Nil	No intervention
				L	Normal	Nil	No intervention
16	16	14 y	Male	R	Ia	Nil	No intervention
				L	Normal	Nil	No intervention
Total: 16 patients, 22 ears. 6 bilateral, 10 unilateral. Concern aesthetic: 7; earphones retention: 3; hearing loss: 1							

Abbreviations: d, days; L, left, R, right; y, years.

**Fig. 2:**
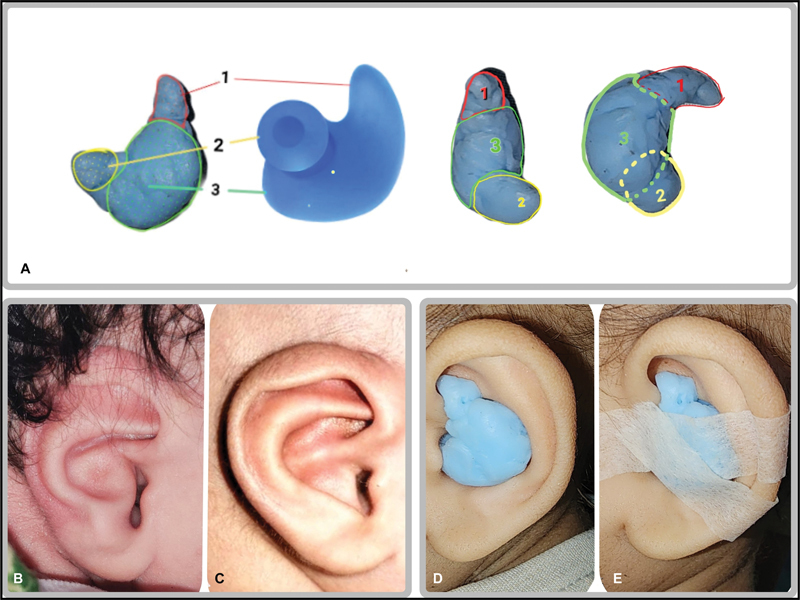
Splint and neonate ear treated with it. ( **A** ) View of the splint made in the shape of an earphone from different angles. Area marked as 1 fits in the cymba concha behind the root of the helix, 2 fits in external auditory meatus, and 3 fits in the conchal bowl. ( **B, C** ) Right ear of a neonate with anteverted concha before and after treatment. ( **D, E** ) Left ear of a patient with splint in situ.

**Fig. 3:**
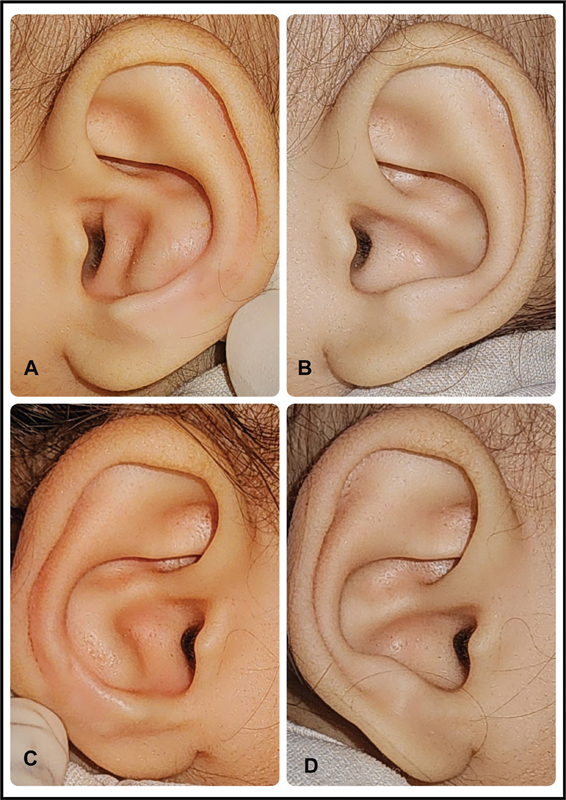
Neonate ear treated with splint. ( **A, B** ) Left ear before and after treatment. ( **C, D** ) Right ear before and after treatment.

**Fig. 4:**
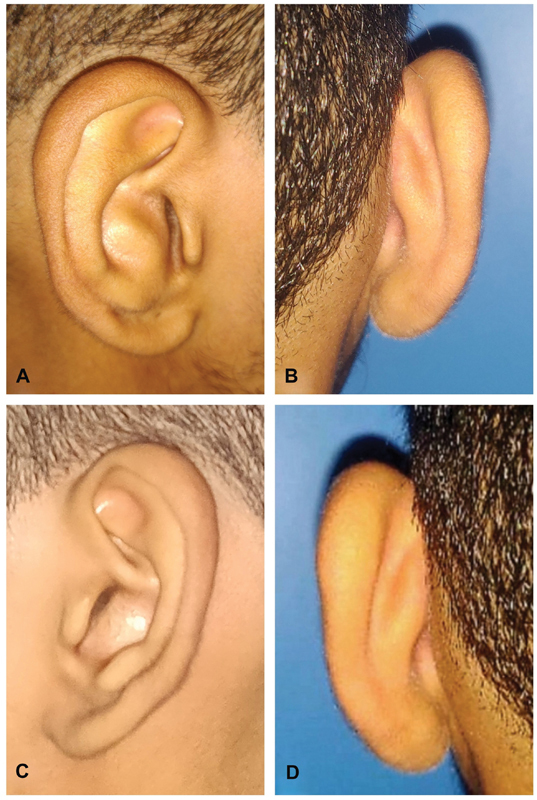
Operated patient pre-op both ears. ( **A, B** ) Right ear front and rear view. ( **C, D** ) Left ear front and rear view.

Three patients sought correction of anteverted conchae. Two neonates underwent noninvasive molding therapy with a custom-made splint ( Figs. 2 and 3 ) and one adult patient underwent surgical correction (mobilizing conchoplasty; Fig. 4 ).

## Technique

The custom-made splint was made of thermoplastic material. The splint was shaped like an in-the-ear earphones ( Fig. 2 ). The splint was used for 23 hours a day for 6 months. The parents were taught about the removal and application of the splint. They were instructed to remove the splints intermittently and watch for any signs of pressure changes on the skin. Their parents were also taught manual molding therapy. It was performed twice daily. Both patients presented to us on the second day of life, and splint and molding therapy was started on the next day, that is, the third day of life. The improvement was noticed as early as 1 week after starting treatment and was significantly corrected by 3 weeks. Maintenance was continued for 6 months. Both the babies showed good correction with no recurrence ( Figs. 2 and 3 ).

**Fig. 5:**
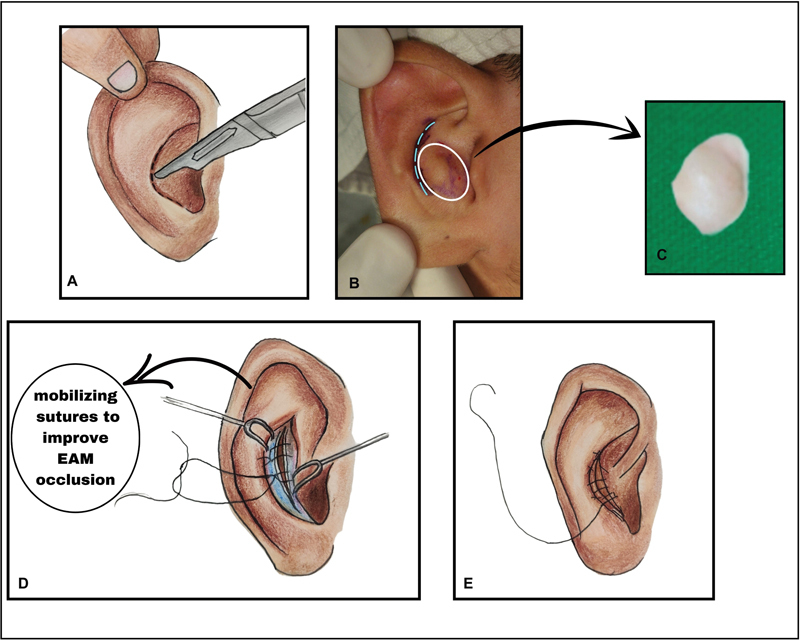
Steps of surgical correction. ( **A** ) Anterior approach. ( **B** ) Markings for skin incision and anteverted conch a. ( **C** ) Excised anteverted part of conchal cartilage. ( **D** ) Mobilizing horizontal mattress sutures between residual edges of cartilage to improve external auditory meatus (EAM). ( **E** ) Skin closure.

The patient who underwent surgical correction was a 17-year-old adolescent boy referred by an otolaryngologist with a presentation similar to keratosis obturans but without any bony obstruction. His cavum concha had convexities in both ears ( Fig. 4 ). Convexity of the cavum concha was more severe on the right side than on the left. The external auditory meatus (EAM) was significantly occluded on the right side because of convex concha. Plugs of desquamated epithelium were present in the right EAM, which was removed preoperatively by an otolaryngologist. He had conductive hearing loss on the right side. Cymba concha was normal in both ears. The skin on the posterior aspect appeared normal with reciprocal concavity, which was observed close to the EAM. The patient had functional complaints pertaining only to the right side. Other features of the external ear, such as helix, antihelix, tragus, antitragus, and lobule were normal in both ears. The patient had no other auricular or facial deformities. The middle and inner ear examinations were within the normal limits in both ears. No other systemic signs or symptoms were noted. The patient sought only right ear correction and did not consent to the procedure on the left side. Excision of the excess and convex conchal cartilage with posterior mobilization of the medial edge of the residual cartilage was performed to reduce the occlusion of the EAM on the right side. We termed this procedure “mobilizing conchoplasty.” The patient was placed in the left lateral position for local anesthesia. Preoperative marking and tattooing of the convex cartilage and an anterior skin incision were performed. Local infiltration using lignocaine (2%) with adrenaline 1 in 200,000 was performed. A skin incision was made 3 mm inside the antihelix (anterior approach; Fig. 5a, b ) and the cartilage was dissected in the subperichondrial plane. Deformed cartilage measuring 1.5 cm × 1 cm × 2 mm was removed, as in the case of the conchal cartilage graft harvest ( Fig. 5c ). Thus, hemostasis was achieved. Posterior mobilizing horizontal mattress sutures were placed between the cut ends of the anteromedial and posterolateral edges of the residual conchal cartilage using 4–0 polypropylene suture ( Fig. 5d ). Skin closure was done using 5–0 polyamide suture ( Fig. 5e ). A conforming dressing with nonadherent gauze was placed over the newly formed conchal cavity, and the external auditory canal was packed. Subsequently, a mastoid dressing was applied. Suture removal was done on postoperative day 10 and a compressive dressing of nonadherent gauze was given. Maintaining corrected EAM patency was considered a priority, and therefore postoperatively, a splint made of thermoplastic material ( Fig. 2a ) was given for EAM to maintain patency. The splint was shaped like an in-the-ear earphone ( Fig. 2a ). An additional firmer splint was given for intermittent use. The splint was advised 23 hours a day for 6 months. The patient was followed up at 1, 3, and 6 month. The patient did not develop any complications.

**Fig. 6:**
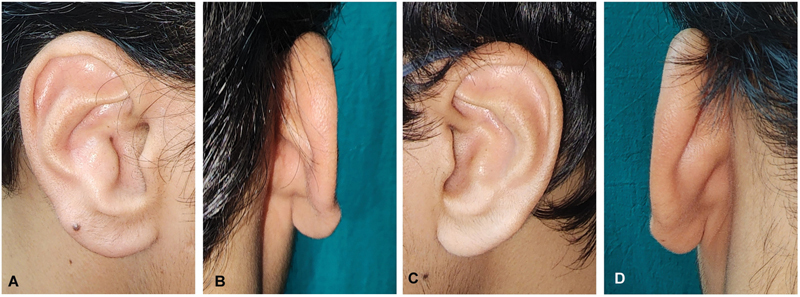
A patient with anteverted concha. ( **A, B** ) Right ear front and rear view. ( **C, D** ) Left ear front and rear view.

**Fig. 7:**
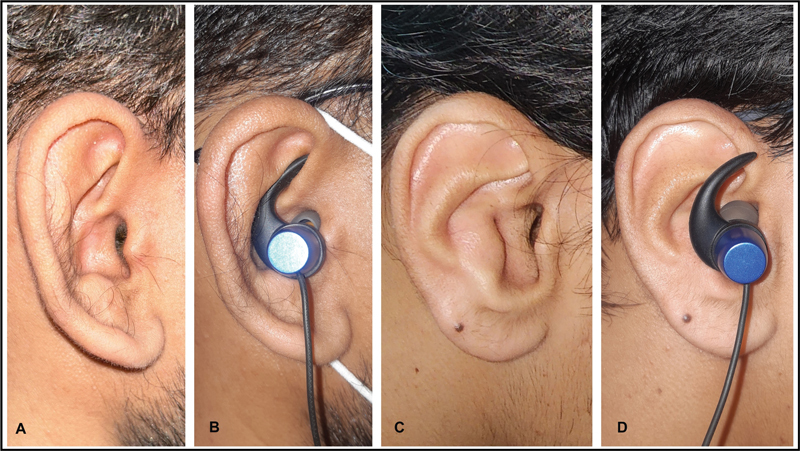
Demonstration of earphone retention issue. ( **A** ) Normal ear. ( **B** ) Normal ear with a well-fitted earphone. ( **C** ) Ear with anteverted concha (AVC). ( **D** ) Ear with AVC with an ill-fitting earphone.

Most of the patients who presented to us were asymptomatic. One patient who underwent surgery experienced hearing loss and cerumen collection. Few had aesthetic concerns but did not seek correction and hence no intervention was made ( Fig. 6 ). Two patients complained of earphone retention issues ( Fig. 7 ).

In addition to reporting these cases, we performed an in-depth literature review. An electronic database search of PubMed, Google Scholar, and the Cochrane Library was conducted on January 2, 2022, using a combination of both Medical Subject Heading terms and plain text related to anteverted conchae. We performed a literature search using keywords anteverted, reverse, convex, or inverted conchae. The manuscripts were reviewed manually by two independent authors (C.S.C. and V.M.M) to identify appropriate studies. Duplicate studies were removed, and the references of the appropriate articles were screened to identify additional related studies. The two authors (C.S.C. and V.M.M.) analyzed and tabulated the data from the selected studies ( Table 2 ). Studies that mentioned an anteverted concha as a part of the Mozart ear anomaly were tabulated separately ( Table 3 ).

**Table 2:** Review of literature (AVC)

Sl. no.	Study	Reported as	No. of cases, no. of ears	Age at presentation	Gender	Side	Symptoms	Category according to our proposed classification	Treatment	Outcome	Comments
1	Yii et al 2	Unusual conchal deformity	1, 2	12 y	M	R	X	IIIa	Surgical. Posterior approach. Skin and cartilage excision	Excellent. No recurrence	
						L	X	IIIa			
2	Shetty et al 10	Reverse (convex) conchal bowl	8, 10	28 y	M	Unilateral	X	X	X	X	
				30 y	F	Unilateral	X	X	X	X	
				32 y	F	Unilateral	X	X	X	X	
				22 y	F	Unilateral	Aesthetic	X	Surgical. Posterior approach. Reversal of convex concha	Excellent. No recurrence	
				52 y	M	Unilateral	X	IIIa	X	X	
				40 y	F	Unilateral	X	X	X	X	
				8 y	M	R	Aesthetic	IIa	Surgical. Posterior approach. Reversal of convex concha	Excellent. No recurrence	
						L		IIIa			
				16 y	F	R	X	IIIa	X	X	
						L	X	IIIa	X	X	
3	Hong et al 6	Congenital inverted conchal bowl deformity	4, 4	3 y	M	R	X	IIa	X	X	Author states that amount of cartilage is not excess but the shape is reversed
				5 y	M	R	Hearing aid retention	IIa	Surgical. Posterior approach. Cartilage not excised. Posterior surface is scored	Excellent. No recurrence	
				3 y	M	L	Cerumen and hearing loss	X	Surgical. Posterior approach. Cartilage not excised. Posterior surface is scored	Excellent. No recurrence	
				7 y	F	R	X	X	X	X	
4	Schönauer et al 1	Anteverted concha	2, 2	3 mo	M	R	Convex shape with apparent EAM obstruction	IIb	Molding with splint changed every 10 d for 10 wk	Excellent. No recurrence	
				32 y	M	R	Aesthetic	IIa	Surgical. Cartilage excised. No sutures to cartilage	Excellent. No recurrence	
5	Osmani et al 4	Congenital inverted conchal bowl deformity	1, 1	33 y	F	L	Earphone retention, aesthetic	Ia	Surgical. Posterior approach. Reversal of convex part of concha	Excellent. No recurrence	This is a rare case of involvement of cymba concha. Cavum concha was normal
6	Scutt et al 12	Anteverted conchal bowl	1, 2	8 y	F	R	Bilateral ear infections and conductive hearing loss, markedly narrow EAM	X	Surgical. Anterior approach. Skin flaps raised, cartilage incised in **T** -shaped manner and mobilized to create concave conchal shape. Additional anchoring sutures of antitragus to conchal base	Excellent. No recurrence	Associated conditions included PDA, CLD of prematurity, GERD, 16p13.11 microdeletion. Post-op CT scan revealed bilateral cholesteatoma within middle ear
						L		X			
7	Baklacı et al 14	Inverted concha	1, 2	25 y	M	R	Bilateral hearing loss and frequent cerumen impaction in his ears, narrow EAM	IId	Surgical. Anterior approach. Cartilage excised. No sutures to cartilage	Excellent. No recurrence	X
						L					
8	Olshinka et al 9	The outward curved concha, an unfamiliar congenital auricular deformation	10, X	All newborns	X	X	X	One photograph shows IIb	Splinting with e EarWell Infant Ear Correction System (Becon Medical, Naperville, IL, United States). A custom-made silicone ear mold (Azoft; Detax, Ettlingen, Germany) was added later to some	Excellent. No recurrence	100% ears corrected by 3 wk. 90% of parents were either extremely satisfied or satisfied
	Total		28, 43						Surgical: 12 Splinting: 21 Nonoperative: 10		

Abbreviations: AVC, anteverted concha; d, days; F, female; L. left; M, male; mo, months; R, right; y, years; X, could not be determined or not mentioned; I, II, IIIa, b, c, AVC classification category.

**Table 3:** Review of literature (Mozart's ear)

Sl. no.	Study	Reported as	No. of cases, no. of ears	Age at presentation	Gender	Side	Symptoms	Category according to our proposed classification	Treatment	Outcome	Comments
1	Gerber20	Mozart's ohr	1,1	X	M	L	Narrow EAM, absent antitragus, square-shaped auricle	X	X	X	X
2	Paton et al17	Mozart ear	2, X	X	X	X	X	IIb	X	X	Two surveys involving a total of 2,277 patients revealing 2 patients of Mozart's ear. Other details are not mentioned. Elucidates first description of Mozart's ears
3	Telich-Tarriba et al8	Mozart's ear	3, 5	9 y	M	R	Inverse concha, slit-like EAM, flattening of the superior crus of the antihelix	IIc	Surgical. Inversion of the convex concha with conchomastoid sutures	At the 1-y follow-up concavity of the concha is maintained but meatal stenosis is present	From the photos, it looks like there is no improvement in EAM obstruction
						L		IIc			X
				9 y	M	R	Inverse concha, EAM stenosis	IIIa	X	X	Associated conditions were CHARGE syndrome, VUR, PN reflux, cleft palate, syndactyly
				5 y	M	R	Inverse concha, EAM obstruction, flattening of the superior crus of the antihelix	IIIb	X	X	Associated conditions were Pierre Robin sequence, cleft palate
						L		IIIb	X	X	
4	Hirose19	Mozart's ear	1, 1	X	X	L	Convex concha, narrow EAM, absent antitragus, square-shaped auricle	IIb	–	–	X
5	García-Cruz et al18	Mozart's ear	1, 2	10 y	M	R	X	X	X	X	Apart from mixed deafness, Mozart's ear, middle and inner ear dysplasia; he had generalized cutis marmorata, a congenital alopecic area on the vertex, right superior esotropia, asymmetric palpebral folds, myopic astigmatism, agenesis of the inferior lacrimal points, micrognathia, malformed ears, Mozart's ear rotated perpendicularly, large hands with clinodactyly of the 5th finger and the equivalent of simian creases, moderate cubitus valgus, a large big toe, shortness of the 4th toe, microgenitalism and bilateral cryptorchidism
6	Yamashita et al3	Mozart's ear	1, 1	13 y	F	L	Earphone fitting, a slit like narrowing of the EAM	IId	Surgical. Posterior approach. Inversion of convex cartilage	6-mo follow-up. Excellent outcome. No recurrence	No associated systemic condition is reported
Total			9, 12						Surgical: 3 Splinting: 0 Nonoperative: 9		

Note: X, could not be determined or not mentioned; I, II, IIIa, b, c, AVC classification category.

## Results

Patients treated with splint and molding modalities showed excellent improvement in ear shape. Improvement was visible as early as the first week of treatment, and reached the optimum shape by the third week of treatment. The correction was maintained until the follow-up duration of treatment, and there was no relapse ( Figs. 2 and 3 ). The parents were satisfied with the improvement in the shape.

**Fig. 8:**
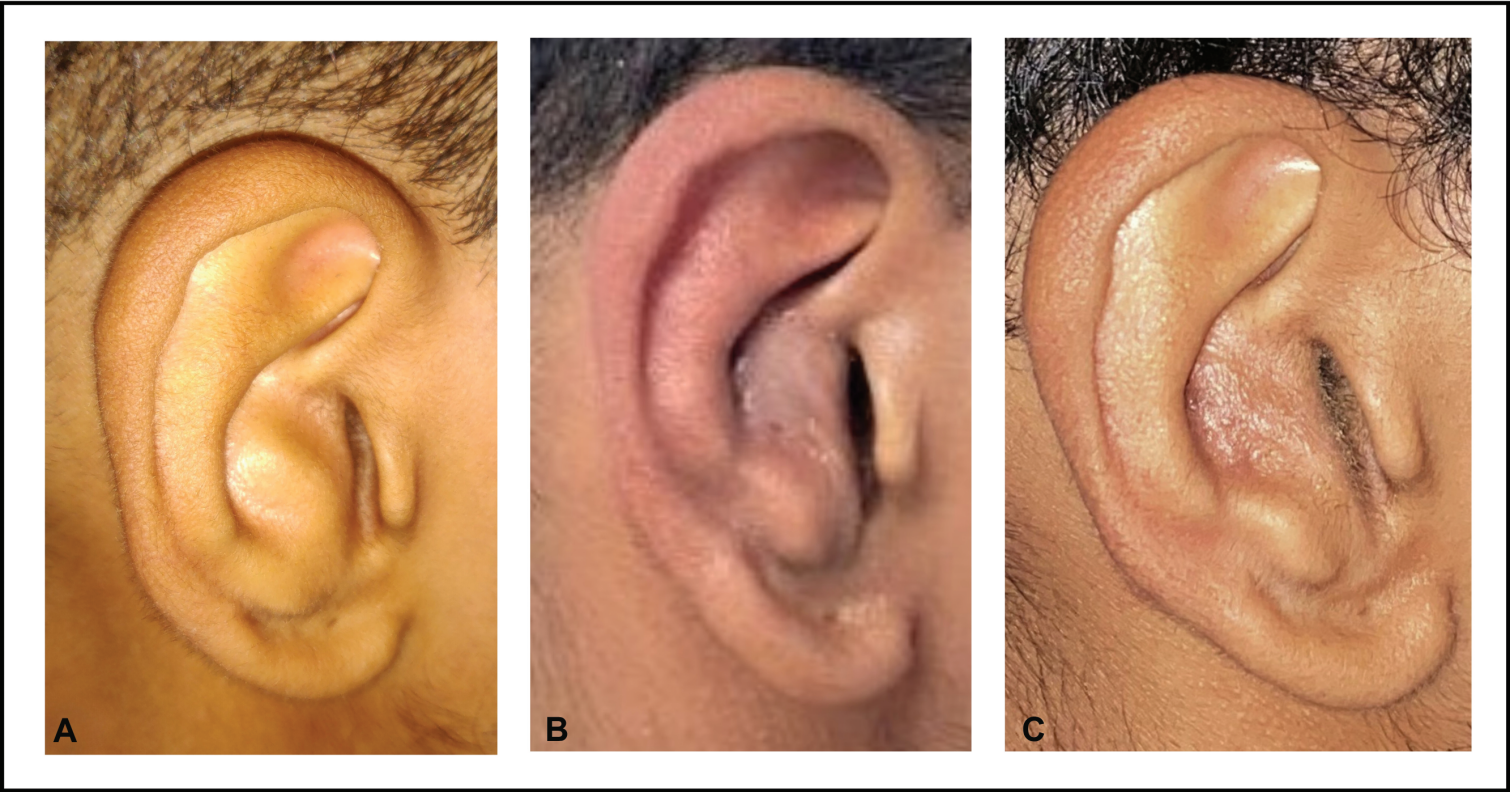
Postoperative outcome of the patient in Fig. 4 . ( **A** ) After suture removal. ( **B** ) After 3 months. ( **C** ) After 6 months. Change to before, at 1 and 6 months.

The patient who underwent surgical correction in the form of “mobilizing conchoplasty” reported significant improvement in hearing loss. The patient's ear shape had improved. The patient was followed up for 6 months, and he maintained the correction, reported no complications, and was satisfied with the outcome ( Fig. 8 ).

## Discussion

The normal shape of the concha is concave. It is termed “anteverted or inverted concha” when it is convex. 2 Yii and Walker first reported this deformity in 1996 and termed it “unusual conchal deformity.” In 2000, Shetty et al described a case series of similar deformity and termed it “reverse or convex conchal bowl.” 10 In 2015, Schönauer et al coined the term anteverted concha. 1 Irrespective of the term used, the shape of the concha was convex instead of a normal concave shape.

Embryologically, anomalies are categorized as either malformations or deformations. 5 Auricular malformations are abnormalities that arise from inherent developmental issues and typically occur between the fifth and ninth weeks of gestation. 6 These malformations can involve deficiencies or excesses in the various components of the auricle. In contrast, auricular deformations occur when external forces disrupt the normal development of the ear structure and can occur at any point during or after gestation. 7

An anteverted concha falls into the category of auricular deformations and can manifest either unilaterally or bilaterally. 3 4 8 9 The deforming forces may result from abnormal muscle vectors caused by aberrant auricular muscle insertion (such as in cryptotia) and/or muscle imbalance (such as in prominent ears) from abnormal positioning in utero or postnatally. 6 The most common deformation involves the helix, resulting in crinkled, Stahl's, or prominent ears. The more rigid central components of the ear, namely, the concha, helical root, and tragus, are less susceptible to deformational forces. 6

**Fig. 9:**
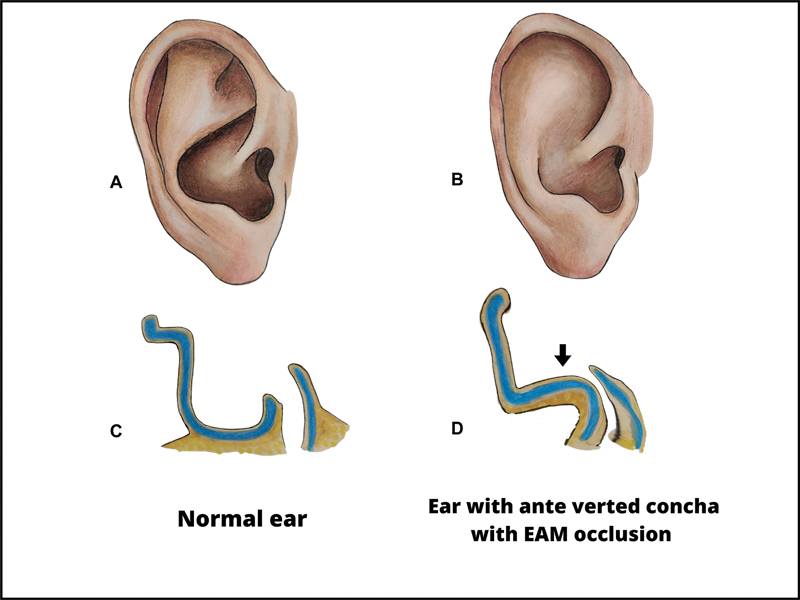
Normal ear and ear with anteverted concha (AVC) causing external auditory meatus (EAM) occlusion. ( **A, C** ) Lateral and cross-sectional diagrams of a normal ear. ( **B, D** ) Lateral and cross-sectional diagram of an ear with AVC that is causing EAM occlusion.

Many of the patients are asymptomatic; however, some experience functional issues. 8 One such issue is the potential development of stenosis in the EAM. When the convexity of the concha is close to the EAC, it creates occlusion of the EAM leading to cerumen impaction and conductive hearing loss ( Fig. 9 ). Additionally, for patients utilizing hearing aids or certain types of earphones, the convex shape of the conchal bowl poses challenges for earmold fittings and retention. Notably, these difficulties tend to be more pronounced in the cases where the entire or a substantial portion of the conchal bowl is affected. Two of our patients had presented with similar earphone retention difficulty ( Fig. 7 ).

The management of anteverted conchae varies depending on the patient's age at presentation. Younger patients are typically treated conservatively with splints or molds to reshape the external ear. This method works best during the neonatal period, when the cartilage is more pliable. However, there is no consensus on a specific age cutoff for splinting. Surgical correction is considered for older patients seeking to correct conchal bowel deformities because of functional problems or cosmetic concerns.

In a newborn ear, because of the influence of maternal hormones, cartilage is soft and malleable, and a splint can be used to correct a variety of deformational problems. Splinting of ear deformities in the early neonatal period has been shown to be a safe and effective nonsurgical treatment for many conditions and is indeed the best treatment for some cases. The anteverted concha certainly falls into the latter group. 1 6 10 15 After a few weeks postpartum, the ear becomes stiffer and less amenable to molding, which makes splinting more difficult, although success can still be achieved with persistence. 7 12 13 16

We performed a literature review and tabulated its findings in Tables 2 and 3 . Table 2 enlists articles that report an anteverted concha. 1 2 4 6 9 10 12 Table 3 enlists articles that have described this anomaly as a part of the Mozart ear anomaly. 3 8 17 18 19 20 The Mozart ear was first described by Gerber in 1898 as a combination of narrow slit-like EAM, absent antitragus, and square-shaped auricle. Later articles considered flattening of the superior crus of antihelix and bulging superior margin of the auricle also as part of the Mozart ear. Not all features were present in all the ears. 3 8 17 18 19

Forty-three ears in 28 patients were reported as anteverted conchae. Of these, 12 ears required surgical intervention, and 21 ears in newborns underwent splinting and molding. The rest did not seek correction. Twelve ears in nine patients were reported as the Mozart ears. Of these, three ears needed surgical intervention. Others did not seek correction. The reasons for seeking correction were EAM occlusion leading to hearing loss, cerumen impaction, earphone or hearing aid retention issues, and aesthetic concerns.

Nonoperative techniques involved splinting, molding, and digital pressure with a variety of materials ranging from dental compound to silicone secured with tapes. Schönauer et al 16 and Olshinka et al 9 have described excellent outcomes in neonates with their nonoperative techniques.

When the cartilage is not amenable to molding and the patient seeks correction, surgical methods are the mainstay of management. Operative techniques for surgical correction include excision of the conchal cartilage without any suturing 2 3 21 and its reverse placement as an autologous graft. 4 14 18 Hong states that the amount of cartilage is not excess, but the shape is reversed. He described surgical correction by scoring the posterior surface of the convex concha. 6 Scutt et al described correction with an anterior approach and involved a **T** -shaped incision of the conchal cartilage and mobilization to a desired shape with anchoring sutures. 12 We believe that while all methods have reported positive aesthetic outcomes, methods that mobilize cartilage will lead to better improvements in EAM occlusion. We believe that in patients with hearing loss, it is necessary to improve EAM occlusion. Cartilage mobilizing techniques described by us as “mobilizing conchoplasty” and “ **T** -shaped incision and mobilization” by Scutt et al 12 are best suited for such patients.

**Fig. 10:**
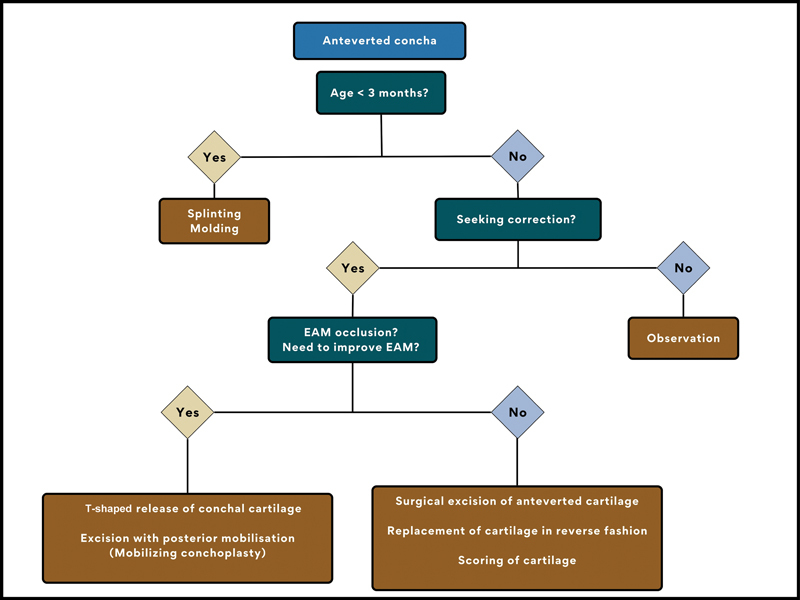
Algorithm for anteverted concha management. EAM, external auditory meatus.

We propose a classification system ( Fig. 1 ) and an algorithm based on it for the management of an anteverted concha ( Fig. 10 ). The classification considers the anatomic location as well as functional complaints of the patients. The algorithm provides suitable options for each subgroup.

We used a thermoplastic splint material to create contoured splints in the shape of earphones. It was secured with a micropore tape. Our operative technique involved not just excision of the convex part of the concha but also mobilization of the anteromedial cut edge toward the posterolateral cut edge, thereby improving EAM occlusion.

A clear limitation of our study is the small number of cases. We believe that with increasing awareness of this deformity, reporting and available data will improve in the future.

## Conclusion

Anteverted conchae may be underdiagnosed. Correction is typically straightforward and may not necessitate surgery if it is diagnosed early. Surgical correction leads to excellent outcomes in patients with symptomatic anteverted conchae. Our proposed classification and algorithm will help further awareness, detection, and standardization of anteverted concha management strategies.

## Compliance with Ethical Standards

Informed consent and assent were obtained before any intervention. All procedures performed in studies involving human participants were in accordance with the ethical standards of the institutional and/or national research committee and with the 1964 Helsinki declaration and its later amendments or comparable ethical standards. Our ethics committee approved this study (IEC-III/OUT/441/2023).
